# Effects of different surgical techniques on mid-distal humeral shaft vascularity: open reduction and internal fixation versus minimally invasive plate osteosynthesis

**DOI:** 10.1186/s12891-016-1224-3

**Published:** 2016-08-26

**Authors:** Zichao Xue, Chaolai Jiang, Chuanzhen Hu, Hui Qin, Haoliang Ding, Zhiquan An

**Affiliations:** 1 Department of Orthopaedic Surgery, Shanghai Jiao Tong University Affiliated Sixth People’s Hospital, No.600 Yishan Road, Shanghai, 200233 China; 2 Department of Orthopaedic Surgery, Ruijin Hospital, Shanghai Jiao Tong University School of Medicine, No.197 Rui Jin Er Road, Shanghai, 200025 China

**Keywords:** Humeral fracture, Reduction, Internal fixation, Blood supply

## Abstract

**Background:**

Humeral shaft fractures are generally managed with the conventional posterior open reduction and internal fixation (ORIF) or minimally invasive plate osteosynthesis (MIPO). This study was aimed at comparing the outcomes of these surgical techniques in terms of the vascular integrity of the mid-distal humeral shaft.

**Methods:**

Twelve upper limbs were harvested from 6 fresh cadavers. ORIF or MIPO was randomly performed on either side of each pair of limbs. The axillary artery was perfused with a latex-lead tetraoxide red solution to visualize the vascular structures. The vascular integrity of the humerus was examined by plain radiography and dissection. The periosteal filling achieved with each technique was scored and the scores compared.

**Results:**

In each limb, one main nutrient artery entering the mid-distal humeral shaft anteromedially (83.3 %) or medially (16.7 %) was first identified. No case of injury to the main nutrient artery was noted for either surgical technique. Injuries to the accessory nutrient arteries entering the mid-distal humeral shaft from the posterior aspect were absent in the MIPO cases, but occurred in 52.9 % of the ORIF cases. In addition, MIPO was also superior to the open plate technique showed superior periosteal filling than.

**Conclusions:**

Our results showed that the MIPO technique is superior to the ORIF in terms of preserving the vascular integrity of the mid-distal humeral shaft.

## Background

Diaphyseal fractures of the humerus are common injuries of the upper arm, and in cases where surgical intervention is necessary, open reduction and internal plate fixation (ORIF) with conventional posterior plating osteosynthesis is considered the best approach [1–3]. However, this approach raises the risks of compromised blood supply and non-union of the fracture due to the associated damage to the soft tissues around the fracture site [2]. Further, ORIF can lead to extensive stripping of soft tissue, disruption of the periosteal blood supply, and iatrogenic radial nerve palsy.

In recent times, a new technique of minimally invasive plate osteosynthesis (MIPO) has been gaining popularity in the treatment of mid-distal humeral shaft fractures [4–7]. In our previous study on 33 patients who underwent MIPO or ORIF, we found that MIPO afforded a lower incidence of iatrogenic radial nerve palsies and more rapid fracture union than ORIF [8]. Studies have also shown that in the case of femoral fractures, MIPO better preserves the vascular integrity of the femur than open reduction and plate osteosynthesis [9]. Considering these findings and the minimally invasive nature of MIPO, we speculated that this technique might also help minimize arterial damage in the case of humeral shaft fractures.

In this study, we applied plates on the intact humeri of fresh cadavers by using either the ORIF or MIPO technique and compared the effects of these techniques on the vascular integrity of the humerus.

## Methods

Twelve upper limbs were harvested from six fresh cadavers (4 male and 2 female) aged 54 to 87 years (mean age, 68.3 years), as available in department of anatomy of medicial school of Shanghai Jiao Tong University. All donors were natural deaths without any history of upper limb trauma arterial thrombosis, or any history of vascular sclerosis, hypertension or nicotine addiction. The limbs were harvested and operated upon within 48 h of death.

### Surgical procedures

The right and left humeri of each cadaver were randomized to undergo either ORIF or MIPO. MIPO was commenced with a 3-cm-long proximal incision made medial to the insertion of the deltoid and lateral to the biceps. Then, the cortex of the anterior humeral shaft was exposed. Another 3-cm-long distal incision was made proximal to the flexion crease, along the lateral border of the biceps. The brachialis was bluntly split to expose the humeral shaft. A submuscular tunnel was prepared and a plate was submuscularly inserted from the distal incision, adjusted to adhere to the anterior aspect of the humeral shaft, and fixed with screws placed distally and proximally [10]. On the contralateral humerus, ORIF was performed by making a conventional posterior longitudinal incision through the triceps, followed by the placement of a plate and its fixation with distal and proximal screws. One author, an attending surgeon, performed all of the surgeries.

### Vascular perfusion

Before the operation, the axillary artery and vein were catheterized and secured with two non-occlusive silk ties [11]. Next, the axillary artery was flushed with 300 mL of warm saline until no blood clots came out from the axillary vein. To ensure optimal results of perfusion, the remaining arterial branches were ligated. After completion of the surgery, the axillary artery was perfused with 150 mL of staining solution (latex: water: lead tetraoxide = 1: 1: 2 in volume) until the dye was extruded through the axillary vein. Thereafter, radiography of the limbs was performed to evaluate the status of the vascular structures. The limbs were refrigerated overnight to harden the dye.

### Vascular dissection

The day after vascular perfusion, all the limbs were dissected anteriorly and posteriorly. An anterior median incision was taken such that it connected the two small incisions made previously. The biceps and the brachial muscle were dissected to expose the brachial artery (the plate was removed if MIPO had been performed). The previously made posterior incision was then reopened to expose the radial nerve and the arteria profunda brachii (the plate was removed if ORIF had been performed). Then, the vascular structures were traced to their origin to assess the integrity of the main nutrient arteries and the accessory nutrient arteries.

### Periosteal filling scores

For evaluation of the degree of periosteal filling, the periosteal vessels were completely exposed and the filling conditions scored by a previously described method [9]. For the purpose of evaluation, the humeral shaft was divided into four zones: proximally fixed portion of the plate, bridging portion of the plate, distally fixed portion of the plate, and bone distal to the plate. Periosteal filling in each zone was separately scored into 4 grades depending on the degree of staining: 0, no staining; 1, mild staining; 2, moderate staining; and 3, marked staining. The difference between the periosteal filling scores of the right and left limbs of each cadaver were calculated to determine the difference between the two surgical techniques. A difference of 9–12 points was considered marked; 5–8 points, moderate; and 2–4, mild; a difference of 0–1 implied that the effects of both techniques were similar.

## Results

### Vascular integrity

**Fig. 1:**
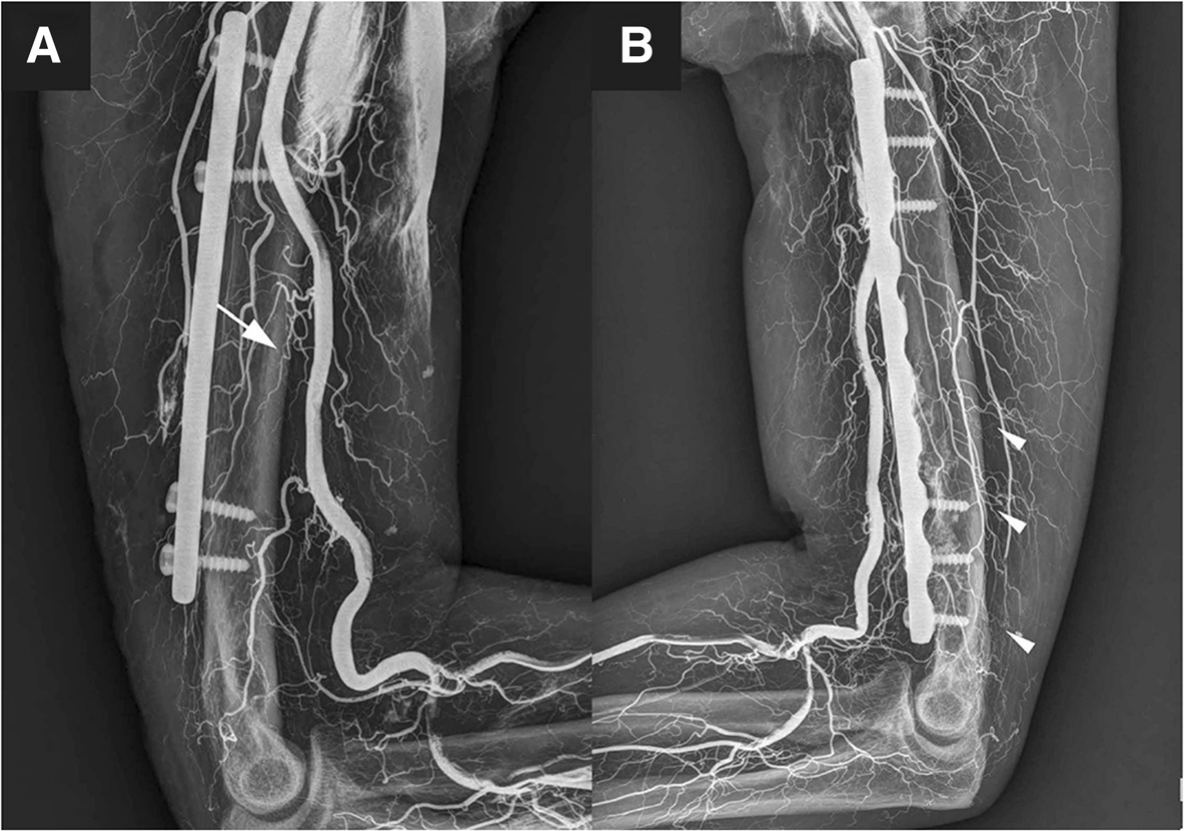
Radiograph images of vascular perfusion. **a** shows the main nutrient artery (denoted by *arrow*) of the humeral shaft, **b** shows the accessory nutrient arteries (denoted by *arrow heads*) of the humeral shaft

**Fig. 2:**
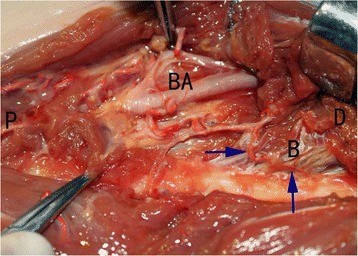
Dissection and observation of main nutrient artery. The integrity of the main nutrient arteries was not affected by either surgical technique. P, the proximal end; D, the distal end; BA, the brachial artery; B, the brachial muscle. The arrows denote the main nutrient arteries

One orthopedic surgeon and one radiologist interpreted the x-ray by a standardized manner to reduce inter-observer variability to the greatest possible extent. Perfusion was successfully achieved in all the 12 limbs. The vascular structure was visualized by plain radiography, as shown in Fig. 1. In each limb, only one main nutrient artery was detected, and in all cases, it originated from the brachial artery and entered the mid-distal portion of the humeral shaft. The main nutrient arteries entered the humeral shaft on the anteromedial aspect in 10 limbs (83.3 %) and on the medial aspect in 2 limbs (16.7 %), and they did not give rise to any branches before entering the humeral shaft. The main nutrient arteries in all the specimens showed good integrity and filling (Fig. 2).

**Fig. 3:**
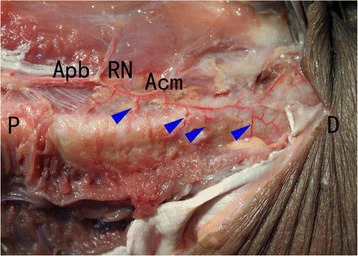
Posterior dissection and observation of specimen underwent MIPO. Accessory nutrient arteries were preserved unaffected by MIPO. Accessory nutrient arteries originated from the deep brachial artery (2–4 branches) and entered the mid-distal portion of the humeral shaft posteriorly, through the radial groove. P, the proximal end; D, the distal end; Apb, the arteria profunda brachii; RN, radial nerve; Acm, arteria collateralis media. The arrow heads denote the accessory nutrient arteries

**Fig. 4:**
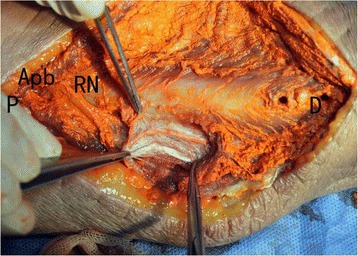
Posterior dissection and observation of specimen underwent ORIF. ORIF damaged accessory mutrient arteries and resulted in profound exudation of the perfusion solution. ORIF also led to very poor periosteal filling condition. P, the proximal end; D, the distal end; Apb, the arteria profunda brachii; RN, radial nerve

Accessory nutrient arteries were found to originate from the arteria profunda brachii (2–4 branches) and enter the humeral shaft posteriorly, through the radial groove (Fig. 3). Specimens in which MIPO was performed showed good preservation of the accessory nutrient arteries in terms of both structural integrity and blood flow (Fig. 3), unlike specimens that underwent ORIF, which showed disruption of both structural integrity and blood flood (Fig. 4) (Table 1).

**Table 1:** The number of damaged accessory nutrient arteries

Cadaver number	MIPO	ORIF
1	0/4	3/4
2	0/3	2/3
3	0/3	2/3
4	0/1	0/1
5	0/4	2/4
6	0/2	0/2
Percentage	0 %	52.9 %

*MIPO* minimally invasive plate osteosynthesis, *ORIF* open reduction and internal fixation

### Periosteal filling

**Fig. 5:**
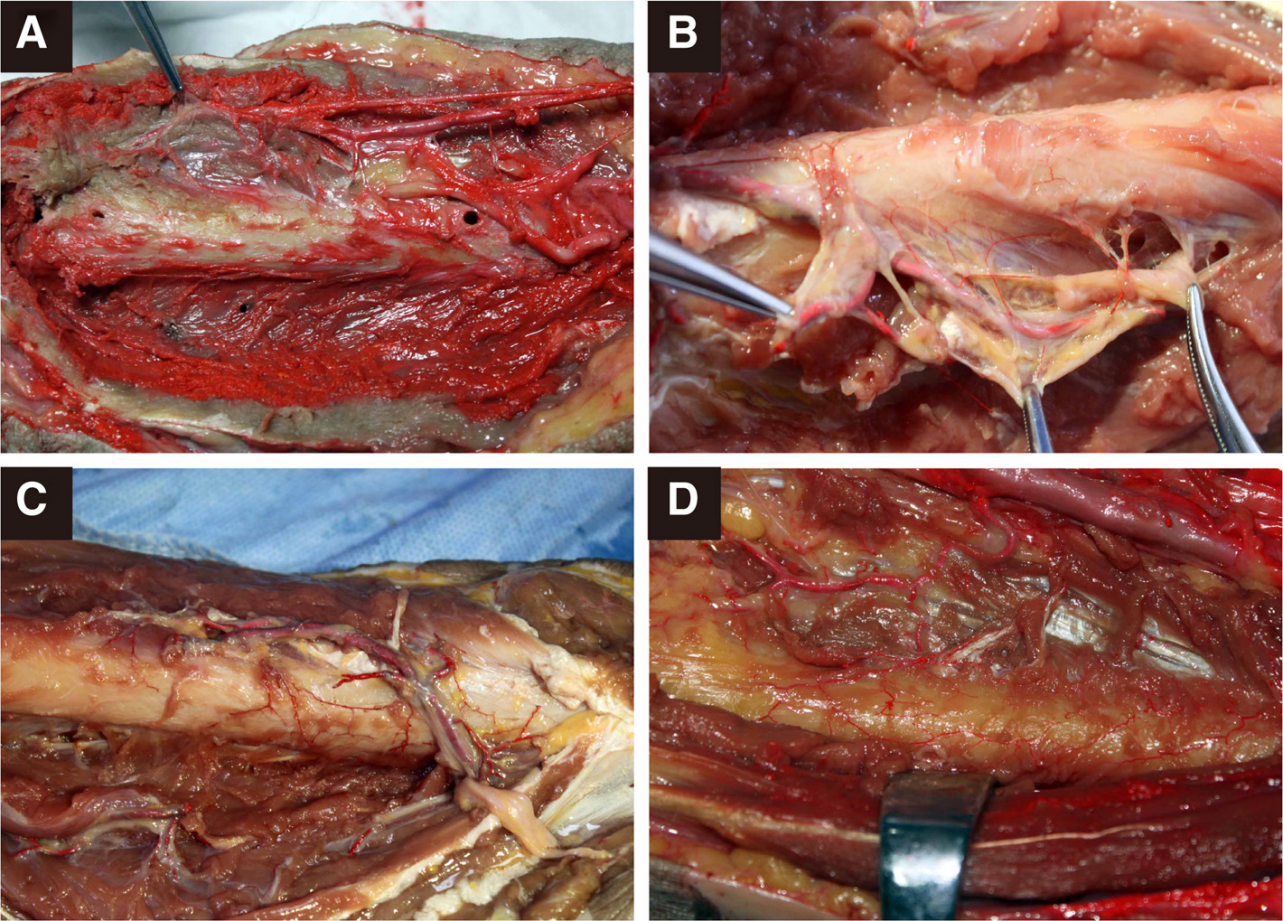
Representative pictures of each grade of periosteal filling. **a**-**d** showed periosteal filling scored as 0–3 respectively

Periosteal filling was evaluated and graded by the method described previously (Fig. 5). As shown in Table 2, the filling and staining of the periosteum achieved with MIPO were better than those achieved with ORIF (Table 2). The differences in the periosteal filling scores obtained for the two surgical techniques were marked in 3 pairs, moderate in 2 pairs, and mild in 1 pair.

**Table 2:** Score of periosteal filling

No	MIPO	ORIF	Score (MIPO - ORIF)
1	12	2	10
2	12	3	9
3	11	4	7
4	11	3	8
5	10	6	4
6	11	2	9

## Discussion

MIPO has been used in the treatment of humeral shaft fractures and has shown some advantages over ORIF [8]. In this study, we sought to determine how these surgical techniques affect the vascular integrity of the mid-distal portion of the humeral shaft. In keeping with previous reports, our study showed that the humerus has one main nutrient artery and several accessory nutrient arteries [12]. Our findings indicated that MIPO was superior to ORIF in maintaining local vascular integrity and promoting periosteal filling at the fracture site.

Neither ORIF nor MIPO affects the stability and functioning of the shoulder and elbow joints, thus allowing early postoperative mobilization and good joint function [13]. In addition, MIPO is also associated with lower risk for iatrogenic radial nerve palsies in comparison with ORIF [4, 8].

Adequate blood supply is essential for the bone union process after fractures [14]. Mid-distal humeral shaft fractures are generally associated with damage to the main nutrient artery of the humeral shaft [15, 16]. Therefore, the blood supply to the fracture site mainly relies on an extraosseous blood supply derived from surrounding soft tissues [17, 18]. However, in the conventional method of open reduction and internal fixation of fractures of the mid-distal humeral shaft, the stripping of the soft tissues and periosteum around the fracture site is unavoidable. This may compromise the poor blood supply to the distal fracture fragments, thereby increasing the risk for non-union. The findings of our study showed that MIPO caused less damage to the accessory nutrient arteries and their blood flow, unlike the case with ORIF, where they were frequently damaged and often necessitated ligation. And it has been confirmed that these accessory arteries is crucial to fracture healing and ligation of them will lead to adverse outcome [19]. Put together, these findings indicate that MIPO might be superior to ORIF in preserving the blood supply of the mid-distal portion of the humeral shaft. And because the fracture pattern is unpredictable, it is preferable to use a minimal invasive approach to preserve the remaining blood supply and minimize the iatrogenic disruption of the perfusion [20].

Some drawbacks of MIPO also need to be considered. Closed reduction required for MIPO is technically difficult; therefore, the surgeon performing the procedure should have received sufficient training and the surgery is prolonged. Further, frequent intraoperative fluorescent examination may be necessary to ensure proper reduction, thereby further extending the operation time. Moreover, angulation deformity is an inherent risk of closed reduction.

This study has some limitations. The two surgical techniques were performed on intact bones, and therefore, the actual impact of these techniques on vascular integrity in the presence of shaft fractures could not be assessed in this study. Furthermore, as the study focused on the effect of different approaches, we did not unify the instruments, among which the screws could have an influence on the integrity of the accessory nutrient arteries.

## Conclusions

Thus, our findings in this cadaveric study showed that the MIPO was superior to the ORIF in preserving the nutrient arteries and periosteal vasculature. This implies that MIPO may help maintain good vascularization of the fracture site, thereby promoting bone union in cases of humeral shaft fractures.

## Abbreviations

MIPO: Minimally invasive plate osteosynthesis
ORIF: Open reduction and internal fixation
